# Changes in Speech-Related Brain Activity During Adaptation to Electro-Acoustic Hearing

**DOI:** 10.3389/fneur.2020.00161

**Published:** 2020-03-31

**Authors:** Tobias Balkenhol, Elisabeth Wallhäusser-Franke, Nicole Rotter, Jérôme J. Servais

**Affiliations:** Department of Otorhinolaryngology Head and Neck Surgery, Medical Faculty Mannheim, University Medical Center Mannheim, Heidelberg University, Mannheim, Germany

**Keywords:** cochlear implant, auditory event-related potentials, speech intelligibility, electroencephalography, source localization, auditory rehabilitation

## Abstract

**Objectives:** Hearing improves significantly with bimodal provision, i.e., a cochlear implant (CI) at one ear and a hearing aid (HA) at the other, but performance shows a high degree of variability resulting in substantial uncertainty about the performance that can be expected by the individual CI user. The objective of this study was to explore how auditory event-related potentials (AERPs) of bimodal listeners in response to spoken words approximate the electrophysiological response of normal hearing (NH) listeners.

**Study Design:** Explorative prospective analysis during the first 6 months of bimodal listening using a within-subject repeated measures design.

**Setting:** Academic tertiary care center.

**Participants:** Twenty-seven adult participants with bilateral sensorineural hearing loss who received a HiRes 90K CI and continued use of a HA at the non-implanted ear. Age-matched NH listeners served as controls.

**Intervention:** Cochlear implantation.

**Main Outcome Measures:** Obligatory auditory evoked potentials N1 and P2, and the event-related N2 potential in response to monosyllabic words and their reversed sound traces before, as well as 3 and 6 months post-implantation. The task required word/non-word classification. Stimuli were presented within speech-modulated noise. Loudness of word/non-word signals was adjusted individually to achieve the same intelligibility across groups and assessments.

**Results:** Intelligibility improved significantly with bimodal hearing, and the N1–P2 response approximated the morphology seen in NH with enhanced and earlier responses to the words compared to their reversals. For bimodal listeners, a prominent negative deflection was present between 370 and 570 ms post stimulus onset (N2), irrespective of stimulus type. This was absent for NH controls; hence, this response did not approximate the NH response during the study interval. N2 source localization evidenced extended activation of general cognitive areas in frontal and prefrontal brain areas in the CI group.

**Conclusions:** Prolonged and spatially extended processing in bimodal CI users suggests employment of additional auditory–cognitive mechanisms during speech processing. This does not reduce within 6 months of bimodal experience and may be a correlate of the enhanced listening effort described by CI listeners.

## Introduction

Cochlear implant (CI) technology has experienced remarkable progress within its 40 years of use and, today, supports a high level of auditory performance for many CI users. However, CI performance still drops substantially in background noise, and even the best performers hear significantly worse than listeners with normal hearing (NH). Moreover, CI performance shows a high degree of variability resulting in substantial uncertainty about the performance that can be expected for any individual CI user. Insufficient knowledge on the time interval, which is needed to reach the individual's maximum speech understanding adds to this uncertainty.

While it is largely unexplored if and how alterations following hearing impairment can be reversed, or how they are compensated with CI use, it is of major interest to identify brain processes related to successful hearing rehabilitation, as well as their time course. Behavioral analysis is not sufficient in this regard. Instead, tools that allow repeated exploration of central auditory processing during the course of auditory rehabilitation are needed. Electroencephalography (EEG) is a useful tool with which to investigate these processes. Because EEG is compatible with the CI device, it allows repeated assessments due to its non-invasiveness, and its high temporal resolution is appropriate for outlining the dynamics of the brain's response to verbally presented speech.

Hearing with an implant, in particular, intelligibility in challenging conditions, needs time to develop, indicating that brain plasticity plays a role (1). In animal models, auditory deprivation is associated with a reduction in connections and a coarsening of the refined connectivity patterns seen with normal hearing (2, 3). In humans, even post-lingual auditory impairment affects processing within the central auditory system as evidenced by loss of lateralization, recruitment of additional brain areas, and cross-modal reorganization (4). As the most obvious improvements in speech understanding are seen within the first 6 months of CI use (5), it is of interest to explore whether brain activity in response to spoken speech changes within this time interval and whether the auditory event-related potentials (AERP) of CI users approximates the responses seen in NH listeners.

Binaural hearing is essential for intelligibility in challenging environments, such as in noisy surroundings (6). Because of extensive binaural interactions in the brain's auditory system, disturbance of input from either ear interferes with central processing of auditory signals (7). Therefore, restoration of binaural input is expected to improve intelligibility, especially in challenging listening conditions. It is unclear, however, whether this can be achieved by current bimodal provision, i.e., electric hearing via CI on one ear and aided acoustic hearing with a hearing aid (HA) on the other ear. Currently, bimodal provision is a common, if not the most common, form of CI provision, but listening remains particularly challenging for this group (8). This may be due to the functional anatomy of the cochlea and the processing characteristics of CI and HA devices, meaning that the electrically and acoustically transmitted signals match poorly regarding frequency representation and timing (9, 10). With bimodal provision, the brain has to combine the divergent signals from both ears and match their neural trace with stored representations of language elements. Extensive auditory training is necessary to achieve this and to adapt to the new set of acoustic–phonetic cues. While there is evidence for a bimodal benefit in speech perception tests (11, 12), neurophysiological alterations associated with successful bimodal comprehension remain to be explored. It is likely that cognitive (top–down) processing compensates for some of the binaural discrepancies in sensory (bottom–up) processing. However, this probably extends and prolongs the brain's occupation with a stimulus (13), which may be disadvantageous for speech understanding. If present, such extensions can be directly evidenced by AERP recordings.

While in typical ecological scenarios listeners are exposed to supra-threshold stimuli, clinical evaluation and much of auditory research is concerned with threshold evaluation, whereas testing of supra-threshold abilities is only at its beginning. When listening to supra-threshold stimuli, problems with intelligibility do arise, specifically in challenging listening conditions such as in background noise. Increasing amplification does not always result in better intelligibility. Therefore, it remains to be explored which processes besides binaural hearing promote supra-threshold intelligibility in noisy environments for CI users (14, 15). Here, again, AERPs may prove to be a valuable tool with which to investigate processes that deviate between NH and CI users and to explore how CI experience changes the brain's response over time.

Bimodal CI users report persistent problems when listening to speech in noisy environments. This is despite ample listening experience. When listening to spoken speech, listeners have to integrate brief and transient acoustic cues, deal with talker variability, and map the auditory input to their mental lexicon, which contains a multitude of partially overlapping word representations. In addition, processing of single words must be rapid in order to be able to follow everyday speech. The processing of spoken speech, from acoustic signal perception to comprehension of meaning, was shown to comprise multiple dissociable steps involving bottom–up sensory and top–down cognitive processing (16–19). It is generally assumed that the mental lexicon of speech elements is retained even during long periods of severe hearing impairment and is still accessible with electric hearing, as evidenced by open set speech understanding in CI users (20, 21). While behavioral measures evaluate the endpoint of this process, AERPs allow continuous recording of the brain's response to speech stimuli and, therefore, are a means to disentangle these processes. AERPs allow exploration of changes to the temporal dynamics of the response during the course of auditory rehabilitation, and to characterize and quantify remaining difficulties. Although natural speech is acoustically complex, AERPs can be recorded in response to natural speech tokens. Responses are stable within an individual, suggesting that they are suitable for detecting changes over time (22). Single steps of language processing have been closely studied by electrophysiological measures in NH and hearing-impaired listeners (16, 19), and they are beginning to be studied in CI users (23–28). Importantly, AERPs of NH listeners represent a template to compare to the responses obtained from bimodal CI users.

Besides the time course of bimodal rehabilitation, mapping of an auditory signal to word/non-word categories is a focus of the present study. This is important for the rapid processing of speech elements, and it is learned early in development (29, 30). During classification of an auditory stimulus as a word, an early N1–P2 response is expected, indicating perception of the stimulus and may be followed by a late N400 response related to lexical access (31). The early N1–P2 response is typically elicited by spectrally complex acoustic signals including words. It can be recorded from CI users and has been shown to be modulated by background noise in NH and CI listeners (22–24, 32). The N1–P2 response consists of a negative deflection, peaking about 100 ms after stimulus onset (N1) and a positive deflection at around 200 ms (P2). Larger N1–P2 amplitudes and shorter latencies of the N1 peak are associated with rising sound intensity (33). After CI implantation, N1 shows rapid improvement and stabilizes over the first 8–15 weeks of CI experience for an auditory discrimination task (26). Furthermore, it has been suggested that the N1–P2 complex can be used to monitor neurophysiological changes during auditory training in CI users (32, 34). In addition, auditory cortex activation is dependent on the learned subjective quality of sounds, evidenced by enhanced and faster early processing of speech sounds compared to their non-speech counterparts, and by the stronger cortical response to familiar than to unfamiliar phonemes (29, 30). Thus, familiarity of the sensory stimulus with the stored representation should lead to a stronger and faster N1–P2 response, and with increasing CI experience, N1 and P2 are, therefore, expected to approximate the response seen for NH listeners.

Beyond sensory processing, speech tokens are subjected to higher-order processing for lexical mapping. Starting at about 200–300 ms and peaking at about 400 ms following word onset, a broad negative deflection is typically seen, termed the N400 (31). This late response is observed in response to all meaningful, or even potentially meaningful stimuli, including written, spoken, and signed words, images, and environmental sounds. The N400 reflects activity within a widespread multimodal semantic memory network, and its amplitude is thought to represent the amount of new semantic information becoming available in response to the auditory input (31, 35). As ease of lexical access reduces this response (31), difficulty in matching the incoming signal with stored representations, such as during effortful listening, may be evidenced by an increase as has been shown previously (36). Therefore, this late negative deflection is expected to be enhanced before CI provision but also with little experience in bimodal hearing, while it is expected to reduce to the magnitude seen in NH listeners with ample CI experience. The N400 is of long duration and does not always appear as a single clearly defined peak in individual-subject averages (37). Some studies were able to differentiate two separate speech-related negativities, termed N200 and N400, whereas such a distinction did not show in other studies (19). Because of discrepancies between studies, we, in accordance with Finke et al. (23, 24), use the term N2 following the recommendation in Luck (38), which indicates that the N2 is a negative deflection following the N1 response.

Neuroimaging studies indicate that increases in listening effort are associated with increased activation in general cognitive regions such as prefrontal cortex (4, 39–41). This is reminiscent of developments seen in healthily aging high-performing individuals, where reduction of perceptual and cognitive abilities is compensated by increased engagement of general cognitive brain areas, such as regions of the attention and salience networks of the brain. This is evidenced by greater or more widespread activity as seen in hyper-frontality and loss of lateralization (42, 43). Perceptual auditory abilities are limited in CI users who also report increased levels of listening effort. Bimodal listening appears to be particularly demanding in this respect (8). Therefore, recruitment of additional brain areas during word/non-word classification is expected for the CI group. It is expected to persist despite CI experience and similar intelligibility across CI and NH groups.

The aim of this study was to characterize the unfolding of lexical access in bimodal CI users and to explore whether it approximates the characteristics seen in NH. Our main interest was to explore whether neural efficacy, indicated by shorter latency and more spatially focused neural activation of the late N2 response, increases with CI experience for difficult listening conditions. The focus was on an early stage of language processing, namely, classification of words, as opposed to acoustically similar complex non-word stimuli. To minimize a confounding influence of age-related central alterations, age of each NH listener was matched to the age of a corresponding CI user. Hypotheses are (i) that magnitude of the N1 response is related to audibility. As loudness is individually adjusted to achieve a set intelligibility criterion, N1 amplitude and latency are expected to be similar across NH and CI listeners and to be stable between pre- and post-CI assessments; (ii) later potentials such as P2 and N2 are expected to deviate between CI and NH groups and they are expected to approximate the NH pattern with increasing CI experience; (iii) based on the familiarity of the words as opposed to the non-words, differences will exist between responses to words and non-words in NH, while they may be absent early after implantation but are expected to increase with CI experience in the bimodal group; (iv) as the task remains effortful for the bimodal CI users, additional cognitive resources are expected to be active to compensate for the distorted signals. This should lead to extended processing of the signals evidenced by prolonged activation in the AERP trace and by increased engagement of attention and salience networks of the brain. As listening effort remains high in the CI group, this type of activation is expected to remain higher than in NH despite extended CI experience.

## Participants and Methods

### Participants

Before initiation, the study protocol was approved by the Institutional Review Board of the Medical Faculty of Mannheim at Heidelberg University (approval no. 2014-527N-MA). Prior to inclusion, each participant provided written consent for participation in the study. Consent was acquired in accordance with the Declaration of Helsinki. CI participants were compensated for their time at test days T3 and T4. NH listeners were compensated at their single visit.

Between 2014 and 2017, study participants were recruited from the patients at the CI Center of the University Medical Center Mannheim. Prospective participants were adults with previous acoustic auditory experience. Inclusion criteria comprised first-time unilateral CI provision, a HiRes 90K implant as chosen by the patient, continued HA use at the other ear, and aged between 18 and 90 years. All patients who fulfilled these criteria were approached for inclusion. Exclusion criteria were assessed during an initial interview (T1) and included the presence of an internal stimulator besides the CI, insufficient knowledge of the German language, and more than mild cognitive deficit, as assessed by the DemTect Test (44). The initial interview, study inclusion (T1), and pre-surgery examination (T2) took place on the same day, usually the day before surgery.

Patients received a CI on their poorer ear, while HA use was continued on the other ear. They left the hospital, on average, 3 days post-surgery. Two to three weeks later, they participated in a week-long in-patient program with first fitting of the speech processor, several fitting sessions, and technical instruction on CI use. Post-implantation assessments T3 and T4 were scheduled for 3 and 6 months post-implantation, respectively. At each assessment, study participants underwent audiometric testing, filled out standardized questionnaires [see Wallhäusser-Franke et al. (11) and below], and underwent AERP recordings. Aspects of hearing and tinnitus in this group apart from AERP recordings were reported previously (11, 45). Independent of the study, between T3 and T4, nine of the participants took part in an in-patient program at a specialized CI rehabilitation clinic, whereas the others used regular out-patient CI rehabilitation services.

Control participants were recruited by word of mouth and from the employees of the University Medical Center Mannheim. Inclusion criteria were German as native language, age-adequate normal hearing, no past or present neurological or psychological problems, and right-handedness. Participants underwent the same screening and performed the same tests as the CI group.

Twenty-seven patients with hearing loss at both ears who planned to undergo unilateral cochlear implant provision were screened. One was excluded because of an exclusion criterion, and the remaining 26 were included in the study. Two discontinued the study following sequential bilateral implantation, one decided that study participation after T2 was too much effort, two discontinued for reasons they did not disclose, and one was excluded because of an exclusion criterion that had not been disclosed before. Reasons for not being included in the AERP analysis was missing AERP recordings at one of the assessments for one participant and left-handedness in another participant, leaving AERP data for 18 participants. Another three participants were excluded because of one incidence of sudden hearing loss in the non-implanted ear associated with Meniere's disease, because of not using the HA at the non-implanted ear at T4 or because of substantial changes in loudness tuning of the HA between T3 and T4. This resulted in 15 participants who contributed data toward the AERP analysis. For demographic details of this group, see Table 1. All study participants were native German speakers and used the NAIDA Q70 speech processor. At T2, 80% used a HA at both ears (Table 1), and at T3 and T4, all non-implanted ears were aided by auditory amplification. HA devices were of different brands and were used with participants' typical daily settings during the course of testing.

**Table 1:** Participant characteristics and stimulation level.

	**CI group (*N*_**CI**_ = 15)**	**NH group (*N*_**NH**_ = 14)**
**Age** Mean ± SD (range) in years	57.67 ± 14.95 (27–78)	57.21 ± 13.69 (24–76)
**Sex** female/male	12/3	12/2
**CI ear** left/right	8/7	
**Years with hearing impairment** Mean ± SD (range)	CI ear: 27.20 ± 18.14 (2–56) HA ear: 24.21 ± 19.01 (2–56)	
**Days between implantation and assessment** Mean ± SD (range)	T2: 2.87 ± 7.24 (1–29) T3: 99.47 ± 18.17 (75–145) T4: 235.47 ± 76.96 (170–427)	
**Lifetime with hearing impairment** Mean ± SD in %	CI ear: 53.72 ± 39.01 HA ear: 24.21 ± 19.01	
**HA use at future CI ear** yes/no	12/3	
**PTA-4** Mean ± SD in dB HL		
**Pre-implantation**	CI ear: 96.03 ± 16.81 HA ear: 68.10 ± 17.99	
**Post-implantation**	CI ear: 46.13 ± 12.37 HA ear: 68.18 ± 18.00	
**T2 SNR** Mean ± SD (range) in dB	15.87 ± 6.90 (7–30)	−2.00 ± 2.39 (−6 to 2)
**T3, T4 SNR** Mean ± SD (range) in dB	10.07 ± 5.51^**^ (1–20)	
**T2 words detected** Mean ± SD in %	69.72 ± 11.46	69.72 ± 13.19
**T3 words detected** Mean ± SD in %	61.06 ± 20.83	
**T4 words detected** Mean ± SD in %	68.00 ± 9.47	
**HADS—Anxiety**	T2: 5.80 ± 4.18; T4: 4.20 ± 3.08	
**HADS—Depression**	T2: 4.53 ± 4.56; T4: 3.80 ± 3.95	
**General health rating**	T2: 2.40 ± 0.99; T4: 2.67 ± 0.98	
**Relevant other health conditions**	9	
**Tinnitus** yes/no	11	

Aided thresholds at 0.5, 1, 2, and 4 kHz were recorded separately for each ear in free sound field using standard audiometric procedures (11), and results were averaged (PTA-4). In the CI group, the signal-to-noise-ratio (SNR) needed to detect 70% of the words in a stimulation block during AERP recordings reduced significantly (

** *p = 0.001) between T2 and T3*.

For each participant who completed the AERP measurement, a right-handed, age-, and sex-matched control with age-adequate normal hearing was recruited. Data of one NH participant was not included because of poor AERP recording. Average hearing thresholds between 0.25 and 10 kHz for both ears of the 14 control participants were 17.93 ± 10.32 dB. Demographics of the 14 NH participants are also presented in Table 1.

### History of Hearing Loss

At inclusion, all CI participants could communicate verbally when using their HA. Six participants reported hearing problems since early childhood, while nine had post-lingual onset of profound hearing impairment. On average, severe hearing impairment of the CI ear existed for half of the lifetime, while hearing impairment at the HA ear had shorter duration (Table 1). Causes for hearing loss were unknown for 73%, were due to sudden hearing loss in two, while one had Meniere's disease, and another participant suffered from Stickler syndrome.

### Acceptance of Bimodal Hearing

Until the first formal appointment at the CI Center of the University Medical Center Mannheim 4 weeks following surgery, participants' mean daily processor use was 11 h. At the end of the study (T4), all but one participant reported combined daily use of CI and HA for more than 8 h per day. CI and HA were always used in combination by eight participants, while the others reported situations during which use of the HA was inconvenient. Most commonly, this occurred during conversations in quiet. On a scale from 0 (no change) to +5 (more content) or −5 (less content), satisfaction with the CI was higher (2.67 ± 1.23) than with the HA (0.8 ± 1.97), or with the combination of both devices (1.73 ± 1.91). Life quality had improved for 10 participants and remained unchanged for the others.

All but one participant had performed CI training with different materials during the week preceding T4 with most (10 participants) training 2–4 h per week. All but two participants cohabitated with at least one other person. Reception of CI use by their peers was perceived as being positive by most (nine participants), interested or curious by the peers of two participants, normal by the peers of one, and mixed by the peers of three participants.

### Health-Related Factors

In addition to hearing status, participants indicated their personal judgment about their general health at T2 and T4 (poor: 0, moderate: 1, okay: 2, good: 3, very good: 4). In addition, mental health was assessed with the Hospital Anxiety and Depression scale (HADS) (46) at these assessments (45).

### Setup

Audiometric testing and AERP recordings were performed within a dimly lit sound booth shielded against electromagnetic interference (IAC Acoustics, North Aurora, IL, USA). The booth was connected with the experimenter's room via a glass window, which, together with a camera in the recording booth, allowed constant surveillance of the participant. During testing, participants sat in a comfortable armchair.

**Figure 1:**
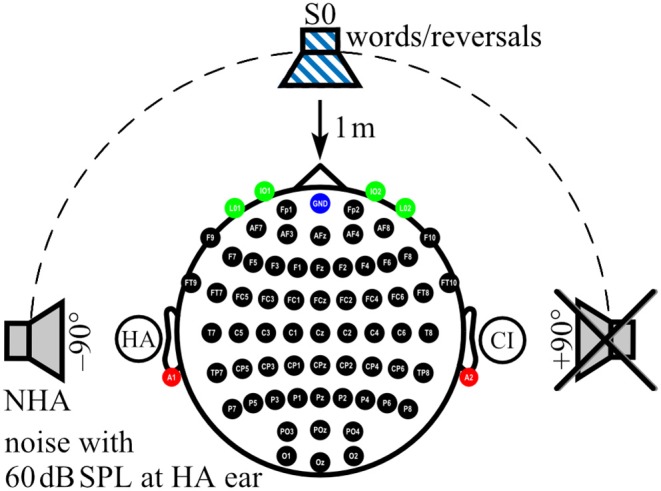
Localization of sound sources and electrode positions. Electrode positions on the scalp (black), ear lobes (red), and eyes (green) are indicated. Ground at Fpz is shown in blue. In the example shown here, a cochlear implant (CI) aids the right ear, whereas a hearing aid (HA) is worn on the left ear. While speech signals were always presented from the front (S0), noise was presented from one of three loudspeakers, here the one facing the HA ear (NHA), whereas the third loudspeaker, here facing the CI ear, was inactive.

During AERP recording and audiometry, auditory stimuli were presented in sound field via an M-Audio Fast Track Ultra USB Audio Interface and BX5 near-field monitor loudspeaker (inMusic Brand, Cumberland, RI, USA) located 1 m in front of the participant (0° azimuth: S0). For noise delivery, two additional loudspeakers of the same type as above were placed at ±90° azimuth at a distance of 1 m to the participant's head (Figure 1). Before each test session, sound pressure level was calibrated by a type 2250 sound level meter (Brüel & Kjær, Nærum, Denmark) with ±0.5 dB accuracy at the center of the participant's head during testing (47).

#### Speech Audiometry

Audiometry performed for this study and self-assessment of the improvement of auditory communication in daily life was described in more detail in a previous report (11). In short, perceived improvements in auditory communication following bimodal provision were assessed with the benefit version of the Speech, Spatial, and Qualities of Hearing Questionnaire (SSQ-B) (48, 49). The questionnaire focuses on speech comprehension (SSQ-B1), localization of sound sources (SSQ-B2), and sound quality (SSQ-B3) in a variety of ecological situations. The reader is asked whether the situation has changed compared to pre-CI hearing. Responses are indicated on a rating scale from −5 to +5. Positive scores indicate improvement, while negative scores indicate worsening, and 0 represents no change. For all questions, there exists the option to tick “not applicable”. Means and their standard deviation were calculated for each of the SSQ-B1-3 scales.

During all audiometric tests, speech signals were presented by male talkers, and speech was always presented in sound field from a loudspeaker in front of the participant (S0). Speech comprehension in quiet was tested with the Freiburger Monosyllable Test (FBE) (50, 51) and the Oldenburg matrix sentence test (OlSa) (52–54). For testing intelligibility in background noise, speech-modulated OlSa noise was presented with a constant level of 60 dB SPL from the front (N0), from the side of the CI (NCI) or the HA ear (NHA) together with the OlSa sentences. Listeners verbally repeated the word (FBE) or each word in a sentence (OlSa) as understood, with the experimenter entering the keywords identified correctly. No feedback was given, and lists were not repeated within sessions. Two lists of 20 words each presented at 70 dB SPL contributed to FBE results, with higher percentages indicating better intelligibility. OlSa sentences consist of five-word nonsense sentences with identical structure and 10 possible words per position. The level of the OlSa speech signal was adapted starting either at 70 dB in quiet or from a signal-to-noise-ratio (SNR) of +10 dB. Twenty sentences were presented per condition with the last 10 contributing to the measure of 50% speech reception threshold in quiet (SRT 50%) or the SNR needed for 50% correct comprehension in noise (SNR 50%). If curves did not show turning points, SRT 50% or SNR 50% for that condition was determined with a second, different OlSa list. In all OlSa tests, lower values for SRT or SNR indicate better intelligibility.

Impact of CI provision on audiometric results was calculated for each audiometric test with the general linear model calculation for repeated measurements (GLM) with Bonferroni correction provided by SPSS24 (SPSS/IBM, Chicago, IL, USA). Values of *p* < 0.05 were considered to be statistically significant, while values of *p* < 0.01 were considered as highly statistically significant. Group means together with their standard deviations (SD) and an indication whether the change between T2 and T4 was significant are shown in Table 2.

**Table 2:** Development of speech comprehension.

	**T2**	**T3**	**T4**	**Significance of change**	**NH**
**FBE** correct in %	57.83 ± 31.45	65.50 ± 25.46	68.17 ± 25.83	*F* = 1.727, *p* = 0.200	98.93 ± 1.62
**OlSa S0** SRT 50% in dB	54.27 ± 17.04	45.77 ± 7.83	43.78 ± 7.18	***F*** = 9.448^**^, ***p*** **= 0.006**	21.25 ± 5.40
**OlSa S0N0** SNR 50% in dB	3.78 ± 5.88	1.17 ± 5.89	0.19 ± 4.56	***F*** = 6.622^**^, ***p*** **= 0.007**	−6.21 ± 2.73
**OlSa S0NCI** SNR 50% in dB	1.91 ± 5.28	0.24 ± 7.49	−0.91 ± 7.47	*F* = 3.132, *p* = 0.059	−12.24 ± 2.21
**OlSa S0NHA** SNR 50% in dB	3.73 ± 5.16	0.92 ± 4.72	−0.67 ± 4.22	***F*** = 9.066^**^, ***p*** **= 0.001**	−12.03 ± 2.72

Intelligibility in the binaural listening condition was assessed before (T2), as well as 3 (T3) and 6 (T4) months post-implantation in bimodal CI users, and for the normal hearing (NH) group. Intelligibility in quiet (S0) was assessed with the Freiburg Monosyllable Test (FBE) (50, 51) at 70 dB SPL and with the adaptive version of the Oldenburg matrix sentence test (OlSa) (52–54) determining the presentation level of the 50% speech reception threshold (SRT 50%). To assess intelligibility in noise, speech-shaped noise was presented from the same source (S0N0), from the side of the CI (S0NCI) or the HA ear (S0NHA) again using the adaptive OlSa method, and the signal-to-noise-ratio (SNR) was determined for 50% understanding (SNR 50%). With bimodal provision, significant improvements (

** *p < 0.01) were seen for sentence understanding in quiet (S0), and with noise presented from the same direction (S0N0) or on the side of the HA ear (S0NHA)*.

#### Data Acquisition

##### AERP

AERPs were recorded from 62 active sintered Ag/AgCl surface electrodes arranged in an elastic cap (g.LADYbird/g.GAMMAcap^2^; g.tec Medical Engineering GmbH, Austria) according to the 10/10 system (55). The electrode at Fpz served as ground (Figure 1). Two additional active sintered Ag/AgCl clip electrodes (g.GAMMAearclip; g.tec) were attached to the left and right earlobes. The electrooculogram (EOG) was monitored with four passive sintered Ag/AgCl surface electrodes (Natus Europe GmbH, Germany) placed below (IO1, IO2) and at the outer canthus (LO1, LO2) of each eye. To protect CI and HA devices, electrodes located above or close to the devices were not filled with gel (mean number of unfilled electrodes: CI: 3, SD: 1.1, range: 1–5; HA: 1, SD: 0.5, range: 0–2) and were interpolated during post-processing. Impedances were confirmed to be below 5 kOhm for passive electrodes and below 30 kOhm for active electrodes. AERP signals were acquired using a 512-Hz sampling frequency by a biosignal amplifier (g.HIamp; g.tec) with 24-bit resolution. Amplifier data acquisition and playback of stimuli were controlled using MATLAB/Simulink R2010a (Mathworks, Natick, MA, USA) with custom MATLAB scripts in combination with g.tec's g.HIsys toolbox. Real-time access to the soundcard was realized with the playrec toolbox (http://www.playrec.co.uk). A trigger box (g.TRIGbox; g.tec) was used to mark stimuli onsets and offsets and to record push button activity (see section on Task and Procedure below) in the continuously recorded EEG data. Stimuli consisted of German monosyllable words taken from the Freiburg Monosyllable Test presented by a male speaker (FBE) (50), which is the clinical standard for speech audiometry in Germany (51). Non-words were generated with the time-reversed audio tracks of these monosyllables (reversals). Only reversals that did not resemble a German word as judged by the lab members were taken as reversal stimuli. Overall, a set of 269 monosyllabic words and 216 reversed words with a mean length of 770 ms (SD: 98 ms, range: 484–1,035 ms) were used for stimulation. Lists with 75 stimuli of which 30% were words and 70% were reversals were generated randomly from the whole set for each stimulation block. Lists were not repeated during an assessment. In addition, speech-shaped noise from the OlSa sentence test (52–54) was presented from a loudspeaker at participants' HA ear or the designated HA ear in NH controls (azimuth ±90°: NHA) at 60 dB SPL. Loudspeaker distance to participant's head was 1 m (Figure 1).

##### Task and procedure

**Figure 2:**
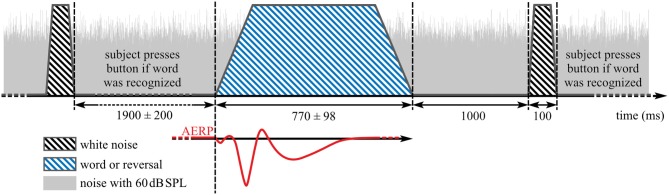
Stimulus presentation during auditory event-related potentials (AERP) recording. Blue striped area: speech stimuli, black striped areas: noise burst that indicates a button press interaction if word was heard before, gray area: background noise with 60 dB SPL.

Participants were instructed to face the loudspeaker in front of them and to keep their eyes closed during recording. In addition, they were instructed to respond only to words by pressing a button after a burst of white noise was played at 75 dB SPL 1,000 ms after offset of each word or reversal stimulus (Figure 2). The test stimuli and white noise bursts were played from the same loudspeaker, similar to the paradigm in Senkowski et al. (56). Inter-stimulus interval between the end of the noise burst and the start of the next stimulus was 1,900 ± 200 ms yielding 75 stimuli per 5 min presentation block (Figure 2). Each presentation block was followed by a short break before the start of the next block. Overall, 4.04 (SD: 0.81, range: 3–7) blocks were recorded per individual assessment.

To avoid ceiling and floor effects, and because intermediate difficulty levels provide the best opportunity for compensatory operation of top–down processes (57), SNR was set to achieve 70% detection of words. This SNR was determined at T2 and T3 in two training blocks, which also served to familiarize participants with the task. If rates deviated substantially from 70% correct classification, the procedure was repeated with an adjusted presentation level. If button press occurred before the noise burst, that particular AERP was excluded from analysis. At T4, two familiarization blocks were administered using the same SNR from T3.

##### Data pre-processing

EEG data were pre-processed offline with MATLAB R2018a (Mathworks, Natick, MA, USA) with the EEGLAB toolbox (version 13.3.2b) (58), and custom MATLAB scripts. Raw data were (1) re-referenced to linked earlobes, (2) low-pass filtered with a cut-off frequency of 64 Hz, and (3) high-pass filtered with a cut-off frequency of 0.5 Hz using finite impulse response (FIR) filters, and (4) segmented into epochs from −300 to 2,200 ms relative to stimulus onset. Epochs with amplitudes in single channels exceeding the threshold range of from −150 to 150 μV were highlighted during visual inspection together with epochs with non-stereotyped artifacts, classified by kurtosis and joint probability (threshold: 3 SD). The final rejection of epochs and identification of poor electrode channels (CI group: Mean: 0.9, SD: 1.5, range: 0–7; NH group: Mean: 0.9, SD: 1.1, range: 0–3) were performed by experienced lab members.

Next, EOG artifacts were removed automatically with a second-order blind identification (SOBI) and independent component analysis (ICA) technique (59–61), as described in Balkenhol et al. (62). To remove electrical artifacts caused by the implant, SOBI ICA was performed. An automated artifact removal algorithm was developed for the present study with identification of artifacts based on their power distribution. Power spectra were determined for all independent components. In response to the acoustic stimuli employed in the present study, implants induced narrow- and wide-band components in the frequency range above 25 Hz. Narrow-band artifacts were automatically detected by a spectral peak search algorithm. Wide-band artifacts were identified by their average power in the frequency range from 40 to 256 Hz, relative to power in the frequency band from 3 to 25 Hz. Thus, if spectral power in the interval from 40 to 256 Hz exceeded power in the low-frequency interval from 3 to 25 Hz, this component was labeled as artifact and removed.

Muscle artifacts, electrical heartbeat activity, as well as other sources of non-cerebral activity were identified by visual inspection of independent component scalp maps and their power spectra (38) and were removed by back-projecting all but these components. Finally, unfilled channels and channels of poor quality were interpolated by spherical splines. On average, 253 ± 58 responses per participant, and assessment remained for data analysis, i.e., 17% of the recorded responses were removed due to artifacts.

##### Data analysis

Data analysis was performed in MATLAB R2018a (Mathworks, Natick, MA, USA) with the fieldtrip toolbox (version 20170925; http://www.ru.nl/fcdonders/fieldtrip) (63) and custom MATLAB scripts. Because optimal ROIs differ between potentials and are uncertain for N2, single-subject averages of all 62 scalp electrodes for the categories “words” (all responses to word stimuli), “reversals” (all responses to reversal stimuli), and the combination of word and reversal stimuli (“all”) were used for N1, P2, and N2 evaluation. AERPs with button press before onset of the white noise burst (Figure 2) were not included in the analysis. For baseline correction, the mean of the pre-stimulus interval from −150 to −50 ms was subtracted from each epoch. The level corresponding to 50% intensity of the stimuli was reached with different delays relative to the stimulus onset. For the analysis of N1, P2, and N2 amplitudes, this delay was corrected by shifting the trigger signal for onset to the first time point the corresponding stimulus reached 50% of its absolute maximal peak amplitude (Figure 2). Mean values in time intervals from 80 to 180 ms, 180 to 330 ms, and from 370 to 570 ms were used as amplitude measures for N1, P2, and N2 (38), while latencies were quantified by the 50% area latency measure according to Liesefeld (64). With this approach, the baseline between two consecutive peaks is calculated by dividing the amplitude difference between these peaks into half. Latency of the later peak is determined by the time point that splits the area below (N1 and N2) or above (P2) this baseline into half. This procedure was also used to estimate the area under the curve.

Statistical analysis was performed with MATLAB's Statistics and Machine Learning Toolbox (R2018a) and custom scripts. Parametric tests were applied to normally distributed data, otherwise non-parametric tests were used. Mean amplitudes, area latencies, and area under the curve of N1, P2, and N2 responses for the categories “words”, “reversals”, and “all” were subjected to separate Dunnett's multiple comparison procedures to compare CI group results at T2, T3, and T4 with the NH group (65, 66). Statistical significance of differences between “words” and “reversals” was explored with *t* or Wilcoxon tests. Values of *p* < 0.05 were considered statistically significant, while *p* < 0.1 was considered to indicate a trend.

##### Source localization

Source localization analysis for the N2 interval was performed with the fieldtrip toolbox and the time domain-based eLORETA algorithm (67, 68). The head model utilized was the standard anatomical magnetic resonance imaging (MRI) data set known as “colin27” (69). Monte Carlo estimates were derived by a non-parametric randomization test (*N*_rand_ = 1000, two-sided) performed with 5 mm lead field resolution on averaged absolute dipole moments. A false discovery rate (FDR) was applied to correct for multiple comparisons.

## Results

### Behavioral Results

Data from 15 bimodal participants contributed to the final analysis (Table 1). Self-assessed improvements of bimodal hearing compared to pre-CI HA-assisted hearing recorded by the SSQ-B questionnaire were largest for speech comprehension (SSQ-B1: 1.42 ± 1.08), lowest for the localization of sound sources (SSQ-B2: 0.91 ± 0.86), and intermediate for sound quality (SSQ-B3: 1.19 ± 1.53). All improvements attained statistical significance (SSQ-B1: *t* = 5.117, *p* < 0.0001; SSQ-B2: *t* = 4.061, *p* = 0.001; SSQ-B3: *t* = 3.023, *p* = 0.009).

Intelligibility in audiometric tests improved with bimodal provision (Table 2) and as reported in Wallhäusser-Franke et al. (11) and Servais et al. (45). Statistically significant improvements were found for OlSa sentences presented both in quiet and within background noise (Table 2). Likewise, the SNR needed to correctly classify 70% of the monosyllabic words in the AERP experiment reduced significantly between T2 and T3 (*T* = 2.758, *p* = 0.001) from 15.87 ± 6.90 to 10.07 ± 5.51 dB (Table 1). For NH, SNR was −2.00 ± 2.39 dB (Table 1).

With the T3 presentation level being retained for T4, the percentage of word identification, as opposed to reversals, was ~70% at T2 and T4, as planned, while average success rate at T3 was 61% (Table 1). Note that the standard deviation is about twice as large at T3 in relation to T2 and T4, indicating increased variability with short bimodal experience. Moreover, SD was much lower in the NH group for all audiometric evaluations (Tables 1, 2).

### AERP

AERPs of the CI group were analyzed regarding differences in CI experience (from T2 to T4) and similarity to NH. The two obligatory evoked potentials N1 and P2 and the event-related N2 potential were analyzed separately, in terms of amplitude, latency, and area, for categories “words”, “reversals”, and the combination of word and reversal stimuli (“all”). Statistical significance of differences was calculated using Dunnett's test, and by *post hoc* comparisons.

#### N1 Response

**Figure 3:**
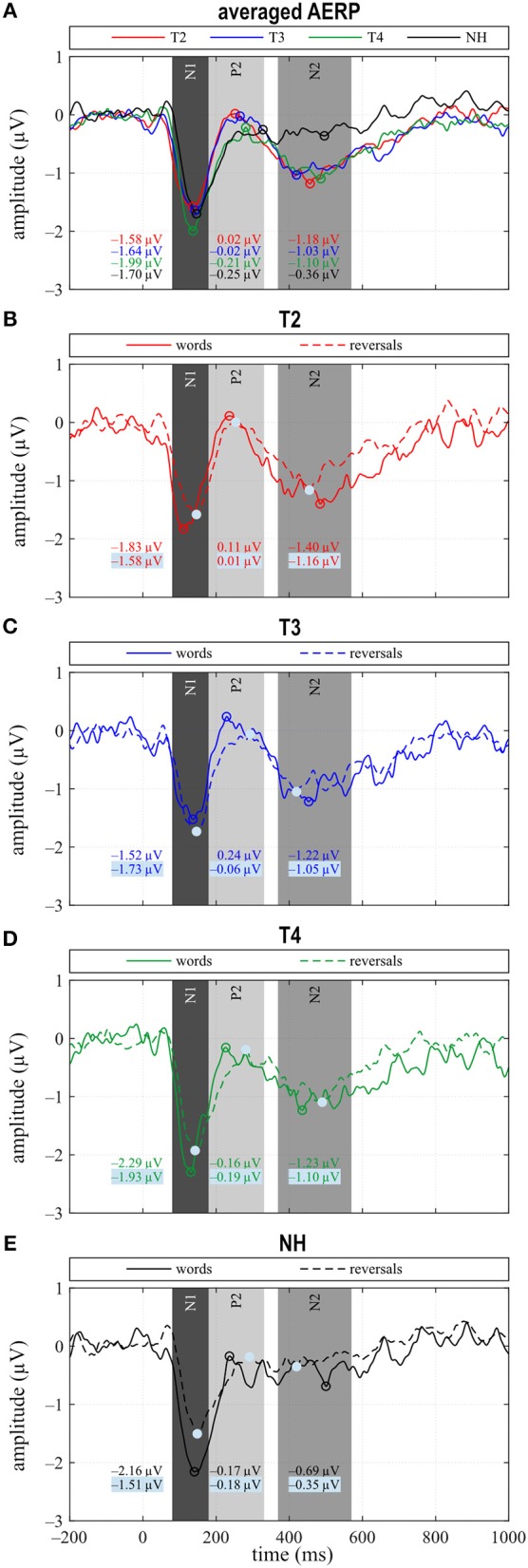
**(A)** Grand averages for all stimuli (“all”) and **(B–E)** for the categories “words” and “reversals”. **(A–E)** Time intervals with N1, P2, and N2 responses are shaded in different grays.

**Figure 4:**
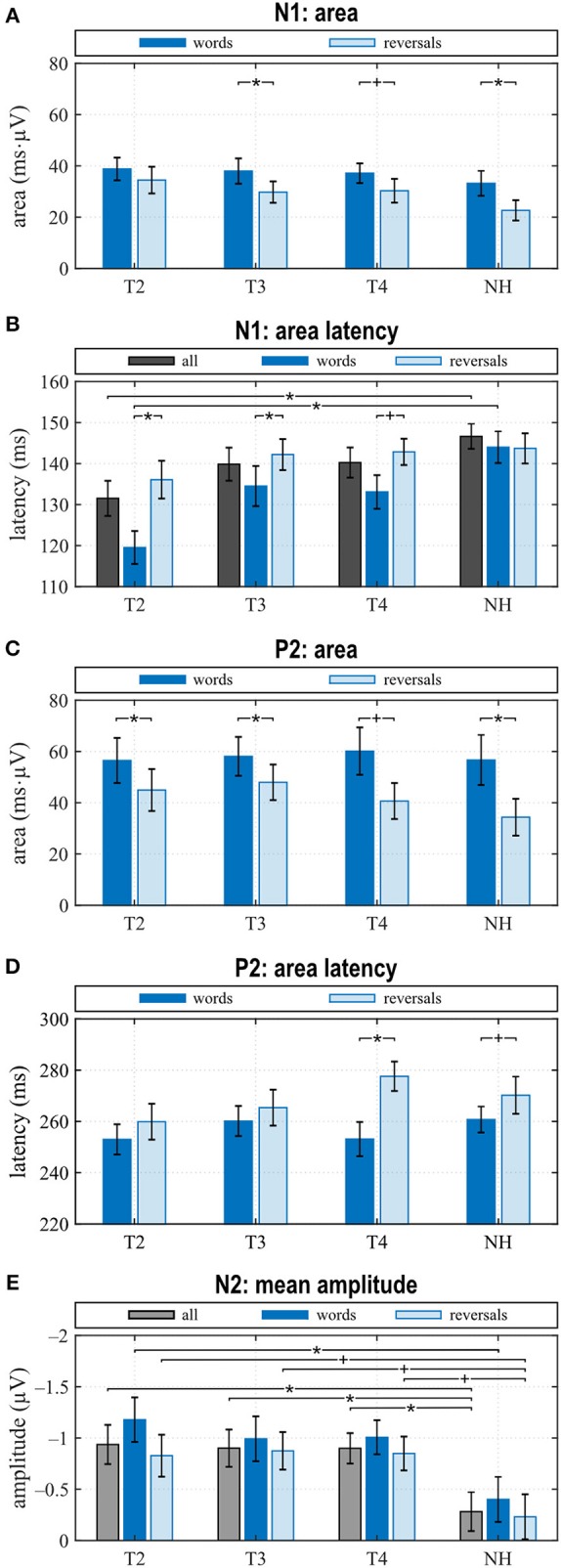
Quantitative AERP results: **(A)** area and **(B)** latency of the N1, **(C)** area and **(D)** latency of the P2, and **(E)** N2 amplitude for the categories “words”, “reversals”, and “all”. **(A–E)** Means with their standard errors; significant differences between conditions are indicated (*p < 0.05 and trends ^+^*p* < 0.1).

N1 amplitude averaged across all stimuli did not differ significantly between groups or between T2 to T4 assessments (Figure 3A), which together with the behavioral results suggests that similar intelligibility across conditions had been achieved as planned. However, in NH, N1 amplitude toward words was significantly larger compared to reversals (*t* = −3.159, *p* = 0.008), whereas this difference, which was largest at T4 (*t* = −1.221, *p* = 0.242) did not attain statistical significance in CI listeners. In addition, N1 area significantly depended on stimulus categories at T3 (*t* = −2.719, *p* = 0.017) and for NH (*t* = −4.180, *p* = 0.001), whereas a trend was evident at T4 (*t* = −1.956, *p* = 0.071) (Figures 3C–E, 4A). Furthermore, data revealed significant differences regarding latency of the N1 depending on group, within-group assessments, and on stimulus categories. A significant main effect was found for N1 latencies in response to words (Dunnett's test: *F* = 5.550, *p* = 0.002) with significantly shorter latency at T2 compared to NH (CI: 119.53 ± 15.543 ms; NH: 143.97 ± 14.44 ms; *p* = 0.0005) (Figure 4B). For the category “all”, *post hoc* testing revealed significant shorter N1 latency at T2 compared to NH (*p* = 0.02), but the main effect showed weak significance (Dunnett's test: *F* = 2.611, *p* = 0.061). Moreover, whereas no latency difference was seen for NH, N1 latencies were significantly shorter in response to words compared to reversals at T2 (*t* = −3.493, *p* = 0.004) and T3 (*t* = −2.201, *p* = 0.045), while a trend was evident at T4 (*t* = −2.080, *p* = 0.056), but significance was lost for T3 after correction for multiple comparisons (Figures 3B–E, 4B).

**Figure 5:**
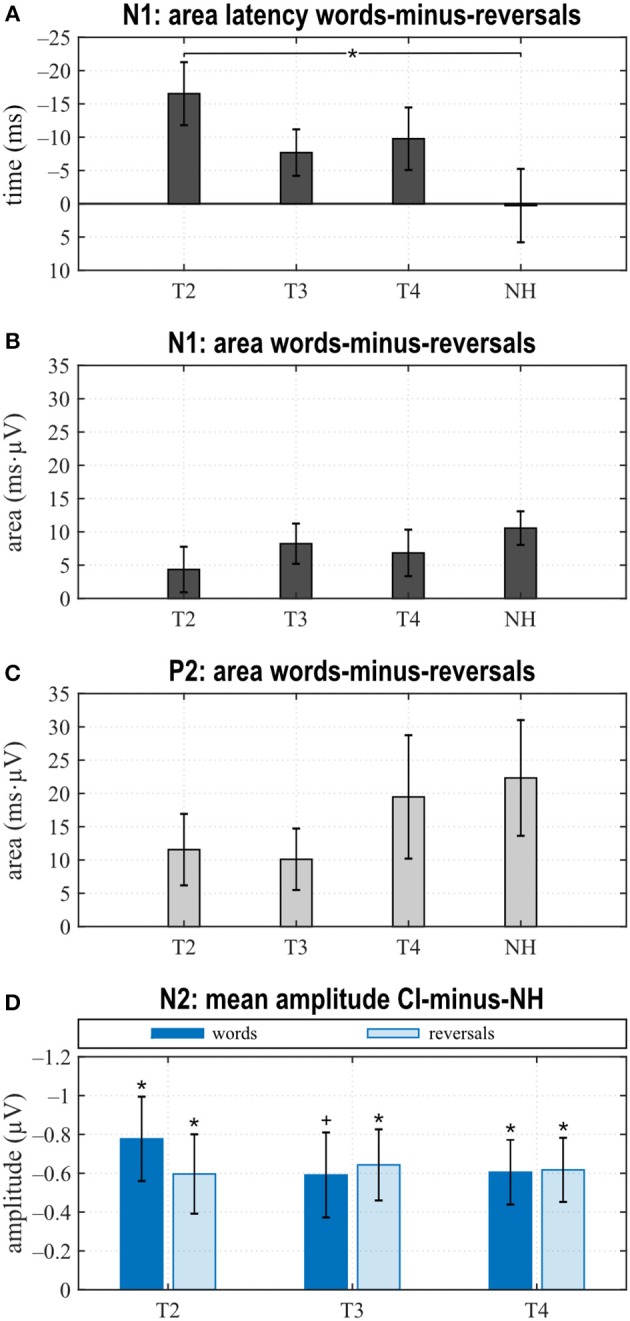
Area latencies **(A)** and areas **(B,C)** of the “reversals” category were subtracted subject-wise from the “words”' category area latency and area results. Dunnett's test revealed significant differences between T2 and normal hearing (NH) for the N1 area latency difference between “words” and “reversals” **(A)**. **(D)** Grand averages of N2 amplitudes for the “words” and the “reversals” categories of the NH group were subtracted from N2 mean amplitudes of individual CI users. Multiple *t* tests revealed significant differences from zero (Bonferroni corrected, *p < 0.0167 and trends ^+^*p* < 0.033). **(A–D)** Mean values and their standard errors are shown.

When comparing differences between responses toward words and reversals, several significant effects appeared. Differences were obtained by subtracting “reversals” latencies/areas from corresponding “words” latencies/areas for each single participant and averaging these for the groups and assessments (Figures 5A,B). Dunnett's test revealed a weak significant main effect for N1 latency (*F* = 2.190, *p* = 0.0996), and *post hoc* testing showed significant differences between the CI and NH group at T2 (*p* = 0.037) (Figure 5A). From T2 to T4, an alignment of the area differences of the CI group with the NH group area differences could be observed for N1 response (Figure 5B). However, Dunnett's tests revealed no significant main effect.

#### P2 Response

In the P2 interval, only responses to words at T2 and T3 had a positive peak, while peak responses at T4, in NH, and toward reversals were negative (Figures 3B–E). At T4, P2 response to reversals was delayed in comparison to words (*t* = −3.674, *p* = 0.003), and a trend for such a delay was present for NH (*t* = −1.794, *p* = 0.096) (Figure 4D). In addition, P2 areas were larger for responses to words than reversals (T2: *p* = 0.049, *t* = 2.154; T3: *p* = 0.046, *t* = 2.188; T4: *p* = 0.054, *t* = 2.101; NH: *p* = 0.023, *t* = 2.568) (Figure 4C).

Area differences were computed as described above. From T2 to T4, area differences aligned to the area difference of the NH group (Figure 5C), and consequently, Dunnett's test showed no significant main effect.

#### N2 Response

The most obvious differences between CI and NH listeners concerned the N2 deflection between 370 and 570 ms after stimulus onset. Whereas a prominent deflection was seen in the CI group at all assessments for both word and reversal stimuli, it was always absent in NH listeners. Therefore, responses toward words and reversals were combined in the category “all”. N2 amplitudes were more negative for the CI group compared to NH, and this difference attained a significant main effect (Dunnett's test: *F* = 3.018, *p* = 0.037). *Post hoc* testing revealed significant differences to NH at all assessments (Figure 4E).

Grand averages of N2 amplitudes for the “words” and the “reversals” category of the NH group were subtracted from corresponding N2 mean amplitudes for individual CI users, and multiple *t* tests showed significant differences from zero for both categories at all assessments (Figure 5D).

### Source Localization

**Figure 6:**
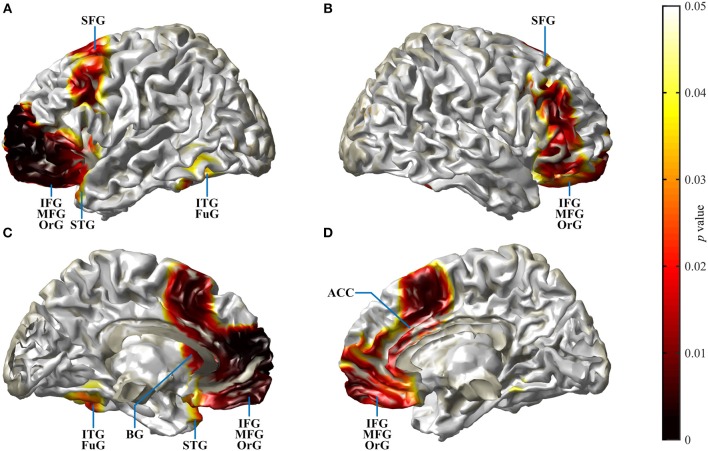
Spatial spread of enhanced cortical activation in CI listeners during the N2 interval. Auditory–cognitive processing is prolonged in CI users in comparison to NH bilaterally in frontal areas [inferior frontal gyrus (IFG), medial frontal gyrus (MFG), superior frontal gyrus (SFG)] and in anterior cingulate gyrus. In addition, in the left hemisphere, significantly enhanced activation is present in inferior temporal gyrus (ITG), at the anterior pole of superior temporal gyrus (STG), and in the rostral basal ganglia (BG). Activity differences between CI and NH listeners are more widespread in the left hemisphere. **(A,B)** View at the left and right hemisphere from the lateral surface. **(C,D)** Left and right hemisphere seen from the midline. For a complete list of CI listeners' brain areas with significantly increased activation, see Table 3. Darkening of the red color scale indicates decreasing *p* values (see scale).

Cortical source localization analysis for the N2 interval was performed with the time domain-based eLORETA algorithm in the fieldtrip toolbox, performing a difference analysis between the CI group at T4 and the NH group. Since the AERP response did not show major differences between responses to words and reversals in either group, the analysis was conducted for the combined word and reversal stimuli (“all”). Increased activation in CI listeners was bilateral but more pronounced in the left hemisphere. Most extensive activation differences were seen in the frontal lobe (Figure 6). Cortical regions, with enhanced activation in the bimodal CI listeners, localized to inferior frontal gyrus (IFG), including Brodman areas BA44, 45, 46, and 47, to orbitorectal gyrus (OrG), and to the medial frontal gyrus (MFG). In addition, extended areas in the superior frontal gyrus (SFG) were more active in CI listeners, comprising areas BA6, 8, 9, and 10. The focus of differential activation in SFG was more dorsal in the left compared to the right hemisphere. Beyond that, enhanced activity in CI listeners was observed in the anterior insula and anterior basal ganglia in the left hemisphere, and bilaterally in the anterior cingulate cortex (ACC: BA24, 32). Finally, a small region in the left inferior temporal and fusiform gyrus (ITG, FuG: BA37) in the temporal lobe showed increased activation. For a complete list of brain regions with enhanced activity in the N2 time window in CI listeners, see Table 3.

**Table 3:** Localization results.

	**Left hemisphere**		**Right hemisphere**	
**Frontal lobe**	**Voxel in ROI**	**% significant**	**Voxel in ROI**	**% significant**
SFG, superior frontal gyrus, medial area BA8	6,770	98	5,961	99
SFG, superior frontal gyrus, dorsolateral area BA8	5,700	55	7,048	43
SFG, superior frontal gyrus, lateral area BA9	7,025	20	6,074	7
SFG, superior frontal gyrus, dorsolateral area BA6	5,314	81	5,394	25
SFG, superior frontal gyrus, medial area BA6	5,970	48	6,191	41
SFG, superior frontal gyrus, medial area BA9	6,895	59	5,589	48
SFG, superior frontal gyrus, medial area BA10	7,535	100	8,193	79
MFG, middle frontal gyrus, dorsal area BA9/46	8,040	20	8,444	42
MFG, middle frontal gyrus, inferior frontal junction	4,609	98	6,362	50
MFG, middle frontal gyrus, area BA46	8,347	83	6,299	7
MFG, middle frontal gyrus, ventral area BA9/46	7,361	67	8,140	92
MFG, middle frontal gyrus, ventrolateral area BA8	6,557	53	7,867	70
MFG, middle frontal gyrus, ventrolateral area BA6	4,982	94	5,010	35
MFG, middle frontal gyrus, lateral area BA10	8,071	94	6,643	46
IFG, inferior frontal gyrus, dorsal area BA44	2,804	92	2,590	32
IFG, inferior frontal gyrus, inferior frontal sulcus	2,666	64	2,980	100
IFG, inferior frontal gyrus, caudal area BA45	2,938	60	2,482	41
IFG, inferior frontal gyrus, rostral area BA45	3,310	93	2,971	100
IFG, inferior frontal gyrus, opercular area BA44	4,501	99	3,790	62
IFG, inferior frontal gyrus, ventral area BA44	2,305	37	2,328	–
OrG, orbital gyrus, medial area BA14	5,044	100	4,001	100
OrG, orbital gyrus, orbital area BA12/47	3,726	94	3,920	90
OrG, orbital gyrus, lateral area BA11	9,471	96	7,518	94
OrG, orbital gyrus, medial area BA11	5,650	93	5,076	98
OrG, orbital gyrus, area BA13	6,243	74	7,364	56
OrG, orbital gyrus, lateral area BA12/47	4,059	97	4,714	100
PrG, precentral gyrus, caudal ventrolateral area BA6	5,556	74	5,832	–
**Temporal lobe**				
STG, superior temporal gyrus, medial area BA38	5,294	46	5,731	–
STG, superior temporal gyrus TE1.0 and TE1.2	5,789	15	6,459	–
STG, superior temporal gyrus, lateral area BA38	5167	50	3,988	7
ITG, inferior temporal gyrus, extreme lateroventral area BA37	1,773	82	2,514	–
ITG, inferior temporal gyrus, ventrolateral area BA37	2,683	59		–
FuG, fusiform gyrus, medioventral area BA37	6,142	52	6,869	6
FuG, fusiform gyrus, lateroventral area BA37	6,989	74	7,926	–
**Occipital lobe**				
MVOcC, medioventral occipital cortex, rostral lingual gyrus	6,954	4	5,975	18
LOcC, lateral occipital cortex, area V5/MT+	6,484	27	5,931	–
**Insula**				
INS, insular gyrus, ventral agranular insula	1,698	98	1,818	17
INS, insular gyrus, dorsal agranular insula	1,968	100	2,109	33
INS, insular gyrus, ventral dysgranular and granular insula	2,174	16	2,188	–
INS, insular gyrus, dorsal dysgranular insula	2,360	52	2,965	–
**Cingulate gyrus**				
ACC, anterior cingulate gyrus, rostroventral area BA24	2,217	91	1,509	73
ACC, anterior cingulate gyrus, pregenual area BA32	3,096	100	3,979	100
ACC, anterior cingulate gyrus, caudodorsal area BA24	2,088	99	3,044	92
ACC, anterior cingulate gyrus, subgenual area BA32	3,250	100	5,063	99
**Basal ganglia**				
BG, basal ganglia, ventral caudate	2,577	73	3,489	15
BG, basal ganglia, globus pallidus	2,558	16	2,571	–
BG, basal ganglia, nucleus accumbens	3,161	30	2,599	2
BG, basal ganglia, ventromedial putamen	2,073	54	2,682	1
BG, basal ganglia, dorsal caudate	5,314	51	4,090	1
BG, basal ganglia, dorsolateral putamen	3,541	16	3,495	–

*List of brain areas with significantly increased activation in the N2 time interval from 370 to 570 ms in CI listeners in relation to NH at T4. Only areas with significantly increased activity of at least 100 voxels are included. The percentage of voxels with significantly increased activity (% significant) in each region is shown separately for left and right hemispheres. Dark gray shading indicates significantly increased activity in at least 75% of the voxels, a white label is used for increased activation in less than 25% of the voxels, and shades in between represent categories 50–75% and 25–49% of the voxels with increased activation. Note that the spatial extent of increased activity is larger in the left hemisphere. If available, Brodmann areas (BA) are indicated*.

### Speech Perception and Brain–Behavior Correlations

Although speech perception improved with bimodal hearing, and this improvement attained statistical significance for three of the five tested conditions (Table 2), speech perception remained worse than in NH after 6 months of bimodal hearing. In quiet, average comprehension was 30% lower for the monosyllable FBE test. Also, at T4, CI listeners required 20 dB higher sound pressure level to understand 50% of the OlSa sentences presented in quiet. With noise presented from the same source (S0N0), at T4, CI listeners' SNR 50% was 6 dB higher with bimodal hearing. This difference increased to 12 dB for lateral noise because in contrast to NH, CI listeners did not benefit from spatial release from masking. Despite the small sample size, a large variability in audiometric performance for the bimodal participants allowed us to examine brain behavior correlations. Correlation analysis was performed between the results of the FBE and OlSa tests and all AERP measures at T3 and T4. As variability was low in the NH group, correlations were not computed for this group.

The most significant correlations between OlSa tests and AERP characteristics were seen at T3. These included latency of the N1 for reversals (S0: *r* = 0.518, *p* = 0.048; S0NCI: *r* = 0.564, *p* = 0.028), latency of P2 for words (S0N0: *r* = 0.728, *p* = 0.002; S0NCI: *r* = 0.644, *p* = 0.007; S0NHA: *r* = 0.600, *p* = 0.018); and N2 latency in response to words (S0NHA: *r* = 0.711, *p* = 0.003). In addition, a significant correlation existed between the area of the N1 for words and S0N0 (*r* = 0.537, *p* = 0.039). At T4, the only correlation with a value of *p* < 0.05 was found for the OlSa test with noise presented to the CI ear (S0NCI) and N2 latency in response to words (*r* = 0.529, *p* = 0.042). However, because of the high number of correlations, significance of these comparisons would never survive a Bonferroni correction.

Bivariate correlations between N2 amplitude at T4 and the change in N2 amplitude between T2 and T4 with self-perceived improvement in everyday auditory communication, assessed via SSQ-B1–3 did not achieve statistical significance but showed a trend for a moderate negative correlation between N2 amplitude at T4 and the improvement of speech comprehension (SSQ-B1: *r* = −0.471, *p* = 0.076) and localization (SSQ-B2: *r* = −0.494, *p* = 0.061) recorded at the T4 assessment.

## Discussion

The study objective was to characterize the temporal dynamics of speech processing in bimodal CI users, to explore whether it changes during the first months of CI experience and whether it approximates the characteristics seen in NH. Moreover, it was of interest to explore at which stage of processing differences occur, depending on familiarity of the stimuli and whether this differs between bimodal and NH listeners. The assumption was that neural efficacy, indicated by an earlier classification of the stimuli, together with shorter duration and a more spatially focused neural activation, increases with CI experience. The task performed required monosyllable word/non-word classification. Intelligibility was impeded by adding speech-modulated noise to the non-CI side, and loudness of the stimuli was adjusted individually to achieve similar intelligibility across groups and assessments. To control for changes in central processing associated with aging, age of NH listeners was matched to individual CI users. Presence of AERPs N1 and P2 at all assessments and with all listening conditions indicates that sound had reached the auditory cortex of our hearing-impaired study participants, suggesting successful amplification and functional integrity of central auditory brain structures and pathways. This is in line with literature that reports sensory components with similar morphologies as in NH in response to acoustic stimulation, even after extended periods of auditory deprivation (23, 24, 26, 70).

Bimodal listeners showed the following developments between T2 and T4 and relative to NH: (1) No difference in the N1 amplitude between stimulus types at T2 and development of a difference with bimodal experience, although to a lesser degree than in NH. In addition, N1 latencies in response to words were shorter than to reversed words (“reversals”) at T2, while no difference existed for NH. The latency difference in CI users reduced until T4. (2) An increase in the P2 amplitude in response to words between T2 and T3 followed by a reduction until T4, together with the development of a latency difference depending on stimulus category that was similar to NH. (3) A sustained N2 deflection irrespective of stimulus type, which did not wear off with bimodal experience and which was absent in NH. (4) Enhanced activity at T4 during the N2 interval localized to extended areas in the frontal and prefrontal lobes, all of which have been implicated in speech processing.

### Importance of Longitudinal Studies

Longitudinal AERP studies following CI provision are important to achieve a better understanding on the magnitude and time course of potential reorganizations in auditory and speech-relevant brain systems. The insights obtained throw light on the chances of auditory rehabilitation and how to make best possible use of these. A related reason for repeated measurements is the observed heterogeneity of hearing-impaired individuals' etiology and time course of hearing impairment, the associated deficits, and CI outcome. To date, only a few studies have investigated changes in sensory processing associated with CI experience (26, 70–72), while longitudinal studies of later potentials are missing altogether.

Longitudinal observations exist for the N1, but allow only limited comparison with our findings because of different stimulus types, task requirements, and listening conditions. Only one study (71) also used sound field acoustic presentation and binaural listening conditions, although participants in this study suffered from single-sided deafness (SSD). Legris et al. (71) probed binaural hearing before and up to 12 months post-implantation with stimuli presented at a constant sound pressure level for all assessments. An increase, although not statistically significant, was seen for the N1 amplitude, but only for CIs implanted on the left side. In contrast, Sandmann et al. (26) and Purdy and Kelly (72) adjusted loudness individually and investigated monaural perception via the CI ear. Whereas, N1 amplitude and latency in response to pure tones did not change significantly within the first 9 months of CI use (72), a significant reduction of N1 latency, together with a significant increase in N1 amplitude, was found in response to complex tones within 4 months of the implant being switched on (26). Finally, because their two participants used a magnet-free CI, Pantev et al. (70) were able to perform repeated MEG recordings during the first 2 years following implantation. Sounds were passed directly to the speech processor of the CI, and loudness was set to a comfortably loud level, which was obviously retained for all measurements. N1m and P2m amplitude of the two CI users increased with CI experience. Hence, results of those studies are not contradictory to the present findings, but because of methodological issues, their findings cannot be compared directly to the present results.

### Adaptation to Bimodal Hearing

#### Early Auditory-Evoked Potentials N1–P2

N1 amplitude in general, and latency after CI provision averaged for the combined responses to words and reversed words (“all”) did not differ between CI and NH group, or between the T2 to T4 assessments in the CI group. This suggested similar audibility across groups and assessments, although only after significant adjustments to the SNR. This finding is in line with the results of a recent study reporting N1 amplitudes and latencies that are similar across NH and hearing-impaired listeners (73) and likewise between CI and NH ears of SSD participants, for words presented in background noise (23), if sensation level was adjusted to achieve similar audibility. A closer look at our NH data revealed significant distinctions in N1 amplitude between responses to the familiar sounds of words and their unfamiliar reversals, with N1 amplitudes for words being larger. While a difference in N1 amplitude depending on stimulus type was absent at T2 for the CI group, i.e., with acoustic amplification and worst hearing ability. Responses of the bimodal listeners approximated the difference seen in NH until T4.

In addition, whereas N1 latencies in NH did not differ between stimulus categories, for the CI group, N1 latencies were significantly shorter for words than for reversals, but this difference reduced with CI experience. It is known that focusing attention on stimuli that are behaviorally relevant, e.g., requiring a response like the button press, influences the N1 response (74). A shorter latency of the magnetic field response M100 to an attended auditory stimulus, compared to the unattended condition, was observed in NH, although this difference failed to reach statistical significance (75). Also, the processing of degraded speech was shown to critically depend on attention (76). In addition, a previous study (77) found that N1 latencies in response to stimuli with different voice onset times were longest in good CI performers, while they were shorter in poor performers and in NH. Thus, shorter N1 latency does not necessarily indicate better sensory processing in CI users.

Further relevant findings regarding sensory processing pertained to P2 amplitude and latency. The most positive peak in the P2 interval reached a positive value only during the pre-implantation T2 assessment, i.e., with worst hearing, while it remained negative for bimodal hearing and in NH. This is in line with the assumption that P2 amplitude is larger in the hearing impaired if the task can be accomplished. Others reported a larger P2 amplitude in the moderately hearing impaired as opposed to NH, which was in line with previous studies cited therein and was interpreted as an indication of effortful listening (78). Furthermore, the auditory P2m response arising from intelligible speech is stronger than that which follows unintelligible speech (79). In contrast, a study comparing monaural electric listening in bilaterally hearing-impaired individuals to monaural performance in NH (23) reports significantly larger P2 areas in NH listeners in response to target words in a word classification task. Thus, P2 amplitude may be influenced by several brain processes, or several components may superimpose, leading to divergent results.

Negativity of the P2 response in the current study is interpreted as an indication that it may be overlapped by a contingent negative variation (CNV) potential, a negative deflection commencing in this time window, which is present if participants prepare for an action in response to a signal (80). Note that participants were required to press a button if the stimulus was classified as a word, but only after an alarm signal, which sounded 1,000 ms after each stimulus. The delayed motor response was necessary to keep the participants alert during the recording, to control for intelligibility, and to avoid interference of auditory and motor responses. A CNV can be expected in this setting, although such a superposition was not reported in a previous investigation that used a similar delay of the motor response to an auditory stimulus in a group of CI listeners (56). Alternatively or additionally, the P2 potential may be overlapped by an early onset auditory evoked negativity, which, supposedly, reflects acoustic–phonological word processing and is observed as early as 150 ms over parietal sites (19). Thus, it appears that only a large P2 peak may show as a positive deflection, while a negative P2 may be due to lower P2 amplitude, a larger CNV, or an acoustic–phonological negativity. This ambiguity cannot be resolved in the present results.

The second finding in this time window concerned P2 latencies, which were longer in response to reversed words than to words. This latency difference was significant for CI users at T4. Additionally, a trend toward longer latencies for reversals existed in NH listeners, suggesting that better hearing is associated with faster processing of the familiar sounds of words in comparison to the unfamiliar reversals. The reversed monosyllable words used in the present study were clearly different from regular words in that they mostly contained sound combinations, which are not present in the participants' mother tongue. Experience with one's own language has been shown to support more efficient processing of phonemes that belong to the native language (30). Latency differences were reported for familiarity as in phoneme or word detection tasks (29, 81), but, in addition, also depend on CI performance (28). Importantly, at T3, with little bimodal experience, P2 latency to words correlates significantly with sentence understanding in the presence of noise (S0N0, S0NCI, S0NHA), with shorter P2 latencies being associated with better intelligibility. Similarly, data by Han et al. (77), who investigated N1–P2 amplitude and latency changes depending on voice onset time, suggested the P2 response to be a more sensitive index of speech perception ability in CI users than the N1 potential. Thus, decreased P2 amplitude and shorter P2 latency to familiar sounds may be associated with better hearing, whereas a stronger response may be a marker of inefficient encoding.

Taken together, findings in this early time window suggest that differences in the processing of speech-relevant auditory stimuli between bimodal and NH listeners already start at the subcortical level. In support, more efficient processing of elements for one's native tongue was evidenced physiologically already at the level of the brainstem (82, 83), and Cheng et al. (84) interpret this to be an indication that long-term lexical knowledge has its effect via sub-lexical processing. Therefore, the present findings indicate that efficient processing of the familiar speech elements may be weakened by prolonged hearing impairment, despite pre-implantation acoustic amplification. Consequently, approximation of the response in bimodal listeners to the N1–P2 morphology in NH suggests that processing of speech elements regains efficacy within the first months of CI use.

#### Lexical–Semantic Processing: Late Event-Related Negativity

In bimodal CI listeners, a prominent negative deflection was present between 370 and 570 ms after stimulus onset irrespective of stimulus type, while it was absent in NH. This response did not approximate the NH response during the duration of the study.

Bimodal CI users report an increased effort when listening in noise. Understanding requires more time and is improved if context is known. In their extended ease of listening model, Rönnberg et al. (57) postulate that whereas speech is largely processed automatically in NH and in favorable listening situations, top–down processing takes on a larger role in challenging listening conditions; such as for bimodal hearing in background noise. It has been reasoned that the perceptual organization of acoustic cues takes place subsequent to the obligatory N1–P2 response (20). Further, a MEG study showed differences in responses to acoustic monosyllabic words and pseudowords that occurred around 350 ms after stimulus onset (85). It is known that categorial perception of speech appears to be highly reliant on top–down processes (86) where many aspects of cognitive control manifest in event-related negativities, typically being recorded when the task requires active participation (34). As we assumed, extended top–down cognitive processing to compensate for the distorted auditory signals, prolonged negativity in the AERP trace in a time window following the N1–P2 response was expected. Our results are in line with this assumption. The bimodal CI users show a prominent N2 irrespective of stimulus category. Neither amplitude nor duration of this response reduces with CI experience in the study interval. In contrast, negativity in this time window was absent in NH listeners, again irrespective of stimulus category. This finding suggests prolonged processing of auditory stimuli by CI users where matching with the mental lexicon is required. This is in line with the results of previous reports, evidencing prolonged duration of negativity in this time window for listening with the CI ear (23, 24). Existing literature shows a stronger adaptation of late AERPs to the activation pattern seen in NH and for good CI performers (4). Absence of this late negativity in NH listeners in the current study may be due to the less demanding word categorization task and to the binaural listening condition.

The late negative-going N2 deflection observed in the current results is largely similar to the N400. In general, the N400 response is elicited by meaningful stimuli, including isolated words, pronounceable non-words or pseudowords (31, 35), and any factor that facilitates lexical access reduces its amplitude (31). In keeping with this, the N400 is larger for meaningless pseudowords than for matched common words (87), and as shown in MEG recordings (79), increased intelligibility reduces it. Finally, Finke et al. (24) could relate prolonged N2 negativity to subjective listening effort, to lower behavioral performance, and to prolonged reaction times. In agreement with this literature, we interpret prolonged N2 activity in the brains of our bimodal CI users as an indication of effortful and attentive processing of speech, suggesting slower lexical access or increased uncertainties in lexical matching, which does not resolve within the first 6 months of CI use.

During acclimatization, CI listeners must adapt to a new set of acoustic–phonetic cues and correlate them to their mental lexicon. Words are identified on the basis of lexical neighborhood, i.e., confusability of the individual phonemes and relations of the stimulus word to other words that are phonetically similar (88). It is assumed that NH listeners encode acoustic cues accurately and compare them to a discrete boundary to obtain sharp categories (13). A study in CI users (89) suggests that categories are less discrete and more overlapping, but may sharpen with experience. When hearing spoken words, NH listeners rapidly activate multiple candidates that match the input, and with more information on the correct word, competitors are rejected. In contrast, in eye-tracking experiments, CI users who experience higher uncertainties during the processing of spoken speech were shown to delay their commitment to lexical items (90).

Taken together, the present AERP results suggest that the processing of speech information by CI users is prolonged and possibly requires more cognitive resources to achieve similar behavioral intelligibility to NH listeners. Moreover, results suggest that while early sensory processing approximates the situation in NH, later lexically related processing does not approximate the NH response during the first months of CI use. It remains to be seen, whether this late negative response is a correlate of listening effort and reduces with additional CI experience, or whether it is a correlate of a fundamentally different processing strategy, which is adopted by CI listeners.

### Extended Spatial Activation With Bimodal Hearing

Since the AERP response in the N2 time window differed between groups but not between stimulus categories, contrasts of activation were calculated by subtracting activity in response to all stimuli in NH listeners from that in CI listeners at T4. Taking this approach, activity in brain areas that are active to the same extent in both groups is subtracted out. Several brain areas exhibited increased activation for the bimodal listeners. Differences were mostly bilateral, although more pronounced in the left hemisphere. Increased activation was present in extended regions of IFG including opercular and triangular parts or Broca's region, and in the MFG in the medial as well as in SFG in the dorsolateral frontal lobe. Beyond that, ACC in the medial frontal cortex, left insula, left basal ganglia, and a circumscribed area in the left caudo-ventral portion of ITG all exhibited increased activation in CI listeners. All regions with increased activation in the CI group were previously shown to be involved in speech processing (4, 16–18, 31, 76, 86, 91–93).

BA44 and 45 in the left IFG are regarded as the core Broca areas (17, 92). IFG contributes to processes involved in accessing and combining word meanings, in particular, in demanding contexts (16), and activity in this region is consistently affected by the contextual semantic fit (31). IFG responses are elevated for distorted-yet-intelligible speech compared to both clear speech and unintelligible noise, while IFG is inactive during effortless comprehension (94). The elevated response to distorted speech in the left IFG was insensitive to the form of distortion, indicating supra-auditory compensatory processes (93). Several studies suggest a functional partition of the IFG with an anterior part driving controlled retrieval based on context, a posterior part selecting between representations (31), and a dorsal part being active during effortful auditory search processes (95). In addition to their IFG, older adults rely on MFG and BA6 activation, which also correlates with comprehension (15, 39).

Distribution of increased SFG activity in CI participants differed between hemispheres with increased activation in BA6, 8, 9, and 10 in the left, and in BA8, 9, and 10 in the right. BA6 is a pre-motor area connected to Broca's area (92), anteriorly adjacent BA8 is involved in the management of uncertainty (96), and BA9 is involved in a number of complex language processes (92), while BA10 is implicated in memory recall and executive functions, as well as in language processing that lacks automaticity (97). In the left hemisphere, a connection exists between the Broca region and the SFG (98), and lesions to the left lateral prefrontal cortex impaired decision threshold adjustment for lexical selection (99). In combination with the left IFG, SFG has been shown to be involved in word selection (100) and with conceptually driven word retrieval (101). Moreover, increased predictability was associated with activation in medial and left lateral prefrontal cortices (94). Beyond that, the left dorsolateral prefrontal cortex is associated with task switching and, together with the anterior insula/frontal operculum and ACC, is a region of the cortical attention systems (102).

Left BA37 in ITG has been implicated in categorical perception of speech (86), and dysfunction of this area leads to word-finding difficulties (92).

The insula is another core region of the language system, which is related to both language understanding and production (92). The processing of degraded speech is associated with higher activation of the left anterior insula (39), and together with Broca's area, the anterior insula was shown to be involved in verbal rehearsal (92, 103). ACC, in turn, is highly connected with the auditory and frontal cortices, and the insula (104), and older adults with impaired hearing expressed higher ACC activity (39). Moreover, AERP measurements with eLORETA source localization indicated greater ACC and MFG activation in the N2 interval during visual presentation of non-words that were associated with increased conflict due to similarity for word representation (105). ACC and insula also are key nodes of the attention and salience networks (102, 106), and there is evidence for a decrease in usage of the attentional network in association with successful performance (107). Whereas, processing of degraded speech is associated with higher activation of the left anterior insula, older adults with impaired hearing expressed higher ACC activity independent of task difficulty and consistent with a persistent upregulation in cognitive control (39, 94, 108). Thus, activation of anterior insula and ACC is interpreted as another indicator of a compensation for degraded auditory input.

In the current study, the two groups under investigation differ with regard to their hearing, but experimental conditions were chosen to allow the same intelligibility for all. Therefore, findings are interpreted in the sense that despite hearing provision and supra-threshold stimulation, more brain resources are required in CI users to achieve the same intelligibility. As extended brain activation has been associated with increased listening effort (24, 39), results suggest that speech understanding remains more effortful for the bimodal CI users despite intensive auditory training. Similarly, increased frontal activation suggests successful compensation of the reduced sensory input in CI users as similar performance is achieved despite better (NH) or worse (CI) hearing. Such effects are in accord with the *decline–compensation hypothesis* (42, 43), which postulates a decline in sensory processing and cognitive abilities during aging accompanied by an increase in the recruitment of more general cognitive areas in the frontal cortex as a means of compensation.

### Bilateral Activation

While increased activation in CI users during the word/non-word classification task was left lateralized regarding the insula and ITG, activation differences in the frontal lobe were mostly bilateral, despite the right-handedness of all participants. This may be due to one or several reasons. First, source localization based on AERP recordings is not as precise as localization with other imaging techniques, and paradoxically, activation of the contralateral hemisphere has been attributed to this circumstance (31). Second, although language is clearly left lateralized in right-handed individuals, several aspects associated with speech activate the right hemisphere in a number of tasks (101). Third, areas with increased activation in the CI group are not those concerned with primary phonological analysis but rather of a domain-general nature (16, 31). Finally, a loss of lateralization has been observed as a compensatory mechanism associated with sensory and cognitive decline (42, 43).

### Limitations

A potential limitation, but also an advantage of our study, is that our CI users used the same CI provision, both in terms of implant and speech processor model being used.

Complex speech signals, but also relatively simple phonemes, evoke multiple overlapping neural response patterns, which differ between different speech tokens and phonemes [e.g., (22, 109)]. We chose to use a large set of monosyllable words and their reversals to avoid habituation and to create a more naturalistic situation, and could show that this approach is successful in producing several separable potentials in NH and CI listeners. In support of our study design, the present study's findings are consistent with several other studies investigating speech perception in NH and CI listeners using natural speech tokens (23, 24, 105).

EEG data offer high temporal resolution, which is mandatory for describing evolution of the brain's response to speech stimuli. They are also remarkably stable within an individual over time (22), which justifies assessing changes in the response following CI provision. However, because of the inverse problem and the need to employ source localization techniques, there is no unambiguous localization of the underlying sources. Therefore, localization data should be interpreted with caution (31, 38).

Finally, as in other studies investigating speech perception in CI users with AERPs, sample size is small, and etiology of the hearing loss is heterogeneous. Therefore, our results do not allow generalization to bimodal CI users, and it would be worthwhile to replicate this study using further participants.

## Conclusions

In sum, there are four main findings from the present study:

(1) With bimodal hearing, intelligibility in background noise improves significantly, indicated by a significant reduction in SNR in the AERP experiment and by reduced intensities for 50% thresholds in sentence comprehension tests.
(2) Differences depending on familiarity of the stimuli occur early, at the level of the N1, with an amplitude difference in NH and a latency difference in CI listeners depending on the stimulus category. Differences are also apparent for the P2 potential, with shorter latencies in response to words for NH listeners. With bimodal experience, morphology of the N1–P2 response in CI users approximates the response seen in NH.
(3) A prominent negative deflection from 370 to 570 ms (N2/N400) following stimulus onset is evident for CI users irrespective of stimulus category, while it is absent in NH indicating that central processing of speech is enhanced and prolonged in CI users.
(4) For the N2/N400 time window, extended activation in CI users is shown in frontal brain areas, suggesting an increased need for cognitive processing to compensate for the degraded auditory speech signal.

## Data Availability Statement

The datasets generated for this study will not be made publicly available for ethical or legal reasons. Requests to access the dataset can be directed to the corresponding author.

## Ethics Statement

The studies involving human participants were reviewed and approved by Institutional Review Board of the Medical Faculty of Mannheim at Heidelberg University. The patients/participants provided their written informed consent to participate in this study.

## Author Contributions

TB designed the computational framework, collected and analyzed the data, and wrote the manuscript. EW-F designed the study, collected and analyzed the data, and wrote the manuscript. NR was responsible for the critical review. JS was responsible for the recruitment and critical review.

### Conflict of Interest

This study was partly funded by the Advanced Bionics AG, Staefa, Switzerland. Advanced Bionics AG manufactures the device under investigation in this study. This does not alter the authors' adherence to all the Frontier policies as detailed online in the guide for authors.
